# NMR Profiling of Milk from Treated Dried off Cows

**DOI:** 10.3390/foods15040770

**Published:** 2026-02-20

**Authors:** Antonella Caterina Boccia, Laura Ruth Cagliani, Dalila Iannone, Roberto Consonni

**Affiliations:** National Research Council, Institute of Chemical Sciences and Technologies “G. Natta” (SCITEC), via A. Corti 12, 20133 Milan, Italy; dalila.iannone@scitec.cnr.it (D.I.); roberto.consonni@scitec.cnr.it (R.C.)

**Keywords:** milk, NMR, metabolite profiles, chemometrics, *Aloe arborescens*, cow

## Abstract

The milk metabolite profiles of dairy cows during the dry-off and peripartum periods were investigated using ^1^H NMR combined with chemometric analysis to evaluate the effects of different dry-off management strategies. Milk samples were collected 14 days before dry-off (**T0**) and 28 days after calving (**T1**) from cows receiving an internal teat sealant combined with intramammary antibiotics (**CTR**), an internal teat sealant alone (**SIG**), or an internal teat sealant associated with dietary supplementation of lyophilized *Aloe arborescens* (**ASIG**). Analysis of both aqueous and organic milk extracts revealed no significant metabolite differences among treatment groups. In contrast, a clear discrimination was detected between samples collected at **T0** and **T1**. Aqueous extracts at **T0** were characterized by higher levels of choline, butyrate, branched-chain amino acids, and N-acetylated compounds, whereas **T1** samples exhibited higher levels of saccharides, citrate, phosphorylcholine, and galactose-1-phosphate. Organic extracts at **T0** showed higher concentrations of conjugated linoleic acids (CLAs) and caproleic acid. These findings indicated that the physiological stage of the cows had a more pronounced impact on milk metabolite composition than the dry-off treatments, with no detrimental effects on milk composition or overall metabolite balance.

## 1. Introduction

Bovine milk constitutes a fundamental component of the human diet and is among the most widely consumed food products globally. Its nutritional value is largely determined by its complex composition, which includes high-quality proteins and a diverse array of carbohydrates and lipids, as well as essential minerals and vitamins [1]. In addition to these macronutrients and micronutrients, milk contains numerous bioactive compounds synthesized and secreted by the mammary gland, including enzymes, hormones, and immunoglobulins [2].

As milk production increases and calving intervals shorten, high daily milk yields at dry-off have become more common, making the dry-off process more challenging and raising the risk of health issues during this period [3,4,5,6].

This phase represents a critical transition in the production cycle of dairy cows, characterized by profound physiological adaptations as the mammary gland shifts from an active lactating state to a non-lactating condition. The process involves extensive mammary tissue remodeling, including epithelial cell turnover, structural reorganization, and functional regression followed by regeneration. Appropriate management of the dry period is therefore essential to promote mammary gland recovery, ensure optimal tissue renewal, and support maximal performance in the subsequent lactation cycle, while minimizing the risk of health disorders during this vulnerable period [7,8].

During this transition period, the cow’s physiological status is profoundly influenced by endocrine and metabolic adaptations, accompanied by alterations in immune function. These changes may predispose animals to the development of metabolic and/or infectious disorders, including mastitis [9]. Maintaining optimal health status during both the dry period and lactation has therefore become a major challenge for the dairy industry.

The use of antibiotics has long been a common clinical practice aimed to prevent bacterial infection and maintain high levels of cow productivity [10]. However, the implementation of Regulation (EU) 2019/6 has introduced substantial restrictions on antimicrobial use in livestock production [11,12,13]. This regulation, which has received broad consumer support due to growing demand for residue-free dairy products, prohibits routine preventive antibiotic treatments and permits their administration only in cases of diagnosed pathological intramammary infection, as determined by somatic cell count evaluation or bacteriological culture.

Consequently, both researchers and dairy producers are increasingly encouraged to investigate alternative strategies aimed at meeting nutritional requirements while preserving physiological homeostasis in dairy cows. In this context, numerous studies have evaluated the effects of dietary supplementation in dairy production systems [14,15].

For instance, diets enriched with nutraceutical compounds have been shown to modulate rumen fermentation dynamics and influence gut microbiota composition. Moreover, such supplementation strategies may exert beneficial effects on immune function and metabolic activity, thereby contributing to improved health status and productive performance during critical physiological stages [16,17].

Vitamin E supplementation has been extensively studied for its role in promoting udder health and reducing the incidence of periparturient diseases. Evidence indicates that the efficacy of vitamin E is dose-dependent, highlighting the need for carefully optimized administration protocols to achieve maximal health benefits [18]. Beyond vitamin E, other nutraceutical interventions have demonstrated significant benefits. Cattaneo et al. [19] reported that dietary supplementation with lyophilized *Aloe arborescens* improved hepatic function, mitigated post-partum inflammatory responses, and reduced the incidence of intramammary infections in cows managed under an antibiotic-free protocol during the dry-off period. Importantly, supplementation also increased milk yield in the subsequent lactation without affecting milk composition.

A well-established and widely adopted strategy to mitigate infection risk during the dry-off period is the use of internal teat sealants. These products, serving as an alternative to prophylactic antibiotics, create a physical barrier that prevents pathogen invasion of the mammary gland [20,21,22]. In line with this approach, Cremonesi et al. [23] investigated the combined effects of *Aloe arborescens* supplementation and topical teat sealant application, or topical teat sealant, with or without antibiotic treatment, on the rumen, rectal, and milk microbiomes during the transition period. Their findings indicated that the polysaccharide-based diet, characterized by anti-inflammatory, immunostimulatory, antibacterial, and antioxidant properties, modulated the composition of the rectal and milk microbiomes, whereas no significant effects were observed in the rumen.

These studies highlight the potential of nutraceutical interventions, alone or in combination with physical protective strategies such as teat sealants, to support health, modulate microbiomes, and enhance productive performance in dairy cows, particularly under antibiotic-restricted management systems.

Based on these results, the present study aimed to investigate the metabolite profiling of milk from dairy cows treated with a teat sealant alone (i), in combination with intramammary antibiotics (ii) after supplementation of lyophilized Aloe arborescens (iii). The experimental period extended from 14 days prior to dry-off (**T0**) to 28 days post-calving (**T1**). Meanwhile, based on the treatment, cows were split into three groups and the collected milk fractions were analyzed by high resolution nuclear magnetic resonance spectroscopy (NMR) and chemometrics. Both aqueous and organic profiles of milk samples were compared to evaluate the synergistic effects derived from the treatments.

NMR has transformed food science research and development by providing un-precedented analytical insights offering a non-destructive, highly reproducible approach capable of capturing detailed molecular profiles. Its broad applicability in assessing chemical composition, quality, and safety makes it a powerful tool for metabolomic studies [24,25]. By providing detailed molecular-level insights into milk composition, NMR enables a precise evaluation of how nutritional supplementation and management interventions modulate milk metabolites, thereby informing strategies to optimize both health and productive performance in dairy cows.

## 2. Materials and Methods

### 2.1. Sampling

Milk samples were collected from twenty-seven multiparous, clinically healthy dairy cows that were randomly allocated into three experimental groups and enrolled in the program. Particularly cows belonged to: **CTR group** (10 cows dried off subjected to standardized antibiotic treatment, along with the application of an internal teat sealant); **SIG group** (9 cows dried-off and subjected only to the internal teat sealant treatment); **ASIG group** (8 cows dried-off and receiving a dietary supplement consisting of *Aloe arborescens*, 200 mL of homogenized leaf daily, administered from 7 days prior to dry-off through 7 days post dry-off, along with the application of an internal teat sealant). The *Aloe arborescens* dosage was calculated to ensure a uniform dry matter intake across all animals. Detailed information regarding cow enrollment, dietary composition and experimental sampling has been previously reported [19]. Rations were formulated in accordance with the National Research Council (NRC) guidelines [26]. Milk samples were collected 14 days before the dry-off period (**T0**) and 28 days after calving (**T1**). The collected milk samples were lyophilized as received and stored at −80 °C until the data acquisition.

### 2.2. Samples Preparation

A biphasic extraction was performed at room temperature in glass vials by adding 500 μL of deuterated water buffered at pH = 7.02 (D_2_O, Merck, 99.96 atom % D, Milan, Italy), 450 μL of deuterated methanol (CD_3_OD, Eurisotop, 99.96 atom % D, Saint Aubin, France) and 900 μL of deuterated chloroform (CDCl_3_, Merck, 99.96 atom % D, Milan, Italy) to 60 mg of lyophilized milk. Samples were vortexed and, after 30 min at r.t., centrifuged at 3500 rcf for 10 min at 4 °C. A total of 600 μL of aqueous and organic fractions were used for NMR analysis.

### 2.3. NMR Data Acquisition and Processing

All NMR spectra were recorded on a Bruker AVANCE NEO 600 spectrometer (Bruker Biospin, GmbH Rheinstetten, Karlsruhe, Germany), operating at 14.07 T, equipped with a 5 mm reverse Z gradient cryoprobe Prodigy, and thermostated autosampler, at 298 K.

For the aqueous extracts, the monodimensional ^1^H NMR experiments were acquired with a modified 1D-NOESY sequence including a low-power water presaturation scheme, introducing an extra relaxation delay. 256 scans over 32 K of data and a spectral width of 8197 Hz, were applied. All spectra were acquired with a total relaxation delay larger than 15 s (D1 = 1.2 s, AQ = 2 s, extra delay = 12 s).

A resolution enhancement function, with a line broadening of 0.5 Hz, was applied before Fourier transformation. All spectra were phased, baseline corrected and aligned with respect the α-glucose anomeric proton of lactose occurring at 5.19 ppm (Bruker Biospin software TOPSPIN 4.1.4 version, GmbH Rheinstetten, Karlsruhe, Germany). All spectra were subjected to intelligent bucketing in the range of 0.50–10.00 ppm according to the resonances assignment, with the exclusion of residual solvents signals between 4.70–5.00 ppm (water), and 3.31–3.34 ppm (methanol). Buckets were normalized with respect to the total integral value using the ACD/Spec Manager (ACD Labs, version 11, Toronto, ON, Canada).

For organic extracts, the monodimensional ^1^H NMR experiments were acquired with 128 scans over 64 K of data, and a spectral width of 8197 Hz. All spectra were acquired with a total relaxation delay larger than 15 s. All spectra were phased, baseline corrected and aligned with respect to the TAG (Triacylglycerol) signal at 4.27 ppm. Spectra were subjected to manual bucketing in the range of 0.20–8.15 ppm according to the resonances assignment, with the exclusion of residual solvent signals between 7.07–7.45 ppm and 2.50–3.10 ppm, and between 3.30–3.40 ppm (water). Buckets were normalized with respect to the total integral value using the ACD/Spec Manager.

Bidimensional ^1^H-^13^C HSQC, and ^1^H-^1^H TOCSY spectra of both aqueous and organic extracts were recorded to confirm resonance assignments.

### 2.4. Statistical Methods

NMR data were imported into SIMCA-P 13.0.3 (Sartorius Data Analytics, Umeå, Sweden) for multivariate statistical analysis. Principal component analysis (PCA), projection to latent structures discriminant analysis (PLS-DA), and orthogonal projection to latent structures discriminant analysis (OPLS-DA) were performed by using “mean centering” for aqueous and “unit variance” for organic extracts respectively, as data pre-treatment. To overcome the randomness of OPLS-DA models, 200 permutations were performed in the permutation test, on the corresponding PLS-DA models.

## 3. Results and Discussion

### 3.1. NMR Spectra Analysis

**Figure 1:**
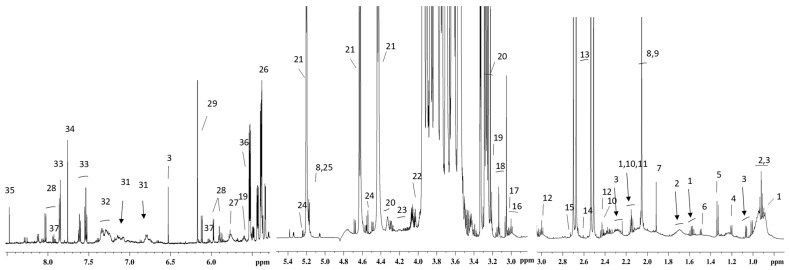
^1^H NMR spectrum of aqueous cow milk extract at 600 MHz and 298 K. Each number in the figure corresponds to a specific identified metabolite: 1, butyrate; 2, leucine; 3, valine; 4, 3-hydroxybutyrate; 5, lactate; 6, alanine; 7, acetate; 8, N-acetylglucosamine; 9, N-acetylcarbohydrates; 10, glutamate; 11, O-acetylcarnitine; 12, 2-oxoglutarate; 13, citrate; 14, methylamine; 15, dimethylamine; 16, creatine/phosphorylcreatine; 17, creatinine; 18, dimethylsulfone; 19, O-acetylcarnitine; 20, glycerophosphorylcoline; 21, lactose; 22, choline; 23, phosphorylcholine; 24, galactose; 25, mannose; 26, glucose 1-phosphate; 27, urea; 28, UDP-derivatives; 29, orotate; 30, fumarate; 31, tyrosine; 32, phenylalanine and derivatives; 33, hippurate; 34, histidine; 35, formate; 36, galactose 1-phosphate; 37, cytidine.

**Figure 2:**
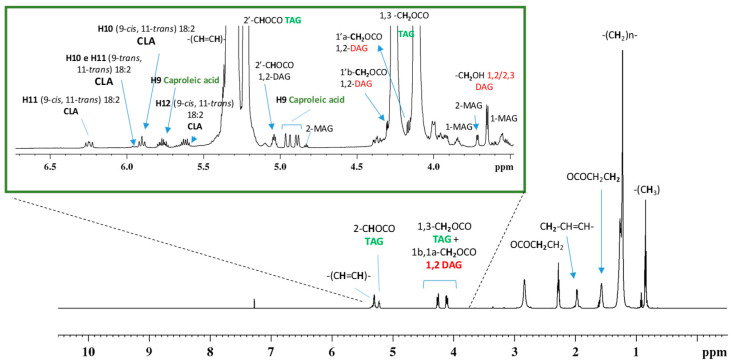
^1^H NMR spectrum of cow milk organic fraction at 600 MHz, in CDCl_3_ at 298 K. The insert shows an expanded region of the spectrum between 3.5 and 6.8 ppm.

^1^H NMR spectra of the aqueous and organic milk extracts are represented in Figure 1 and Figure 2 respectively. The resonance assignment was performed with the aid of bidimensional homo- and hetero-nuclear experiments, and with the use of databases (https://www.hmdb.ca/ (accessed on 5 February 2026); CHENOMX NMR suite V12.0; https://bmrb.io/metabolomics (accessed on 5 February 2026)) and compared with data from the existing literature [27,28,29,30].

#### 3.1.1. Aqueous Milk Extract

The ^1^H NMR spectrum, representative of an aqueous cow milk extract, is shown in Figure 1, with the following identified metabolite numbering.

Milk profiling enabled the identification of several chemical classes, including saccharides, amino acids, organic acids, short-chain fatty acids, nucleotides, alcohol derivatives, organosulfur compounds, and amides, in agreement with previous studies [28,31,32,33,34,35]. Lactose and citrate are the predominant metabolites, as expected, but lower-intensity peaks attributable to less abundant metabolites were also assigned.

#### 3.1.2. Organic Milk Extract

The ^1^H NMR spectrum of organic extract, represented in Figure 2, shows two distinct groups of signals: The first group is dominated by resonances corresponding to the primary components of milk, namely saturated and unsaturated triacylglycerols (TAG) as expected. The methyl and methylenic groups of fatty acid (FA) were assigned at 0.84 ppm and 1.21–1.26 ppm, respectively. The α- and β-methylenic protons of all acyl chains of FA were at 2.27 ppm and at 1.58 ppm respectively, while the unsaturated proton resonances were found in the 5.30–5.34 ppm range. All the remaining glycerol signals were respectively assigned at 4.11 ppm as *sn*-1 and *sn*-3 Ha protons, CH_2_ TAG; at 4.26 ppm as *sn*-3 and *sn*-1 Hb protons, CH_2_ TAG, and finally at 5.23 ppm *sn*-2, CH TAG [36].

The second group of resonances consists of less intense signals indicative of minor constituents, identified by the analysis of two-dimensional experiments and according to literature data [30,36,37,38,39,40]. The unsaturated region of CLA (18:2 conjugated linoleic acid) evidenced the 9-*cis*, 11-*trans*, also known as rumenic acid, as the most prevalent isoform and in less abundance the isomer 9-*trans*, 11-*trans*. In the same region the caproleic acid (C10:1 *cis*-9) due to the characteristic pattern of resonances respectively at 5.75 ppm and 4.94 and 4.88 ppm was identified [40]. Diacylglycerol (1,2-DAG) was also identified by signals at 5.03 ppm and 3.65 ppm; while diacylglycerol (1,3-DAG) was assigned at 3.98 ppm. Monoacylglycerol (1-MAG) were assigned due to the characteristic pattern of signals at 3.52 and 3.61 ppm, and 3.84 ppm. Analogously, 2-MAG were assigned due to the resonances at 3.71 and 4.82 ppm, in the proton spectrum [41]. Butyric acid was also identified for the presence of a characteristic triplet at 0.91 ppm [32]. Cyclopropane fatty acids were identified due to the characteristic high field resonances respectively at −0.37 and 0.54 ppm in the proton and TOCSY spectra (Figure S1) [42]. Finally, linolenic and linoleic acid were also identified due to the multiplets at 2.78 and 2.77 ppm respectively [43].

### 3.2. Multivariate Statistical Analysis of NMR Data

#### 3.2.1. Aqueous Milk Extract

**Figure 3:**
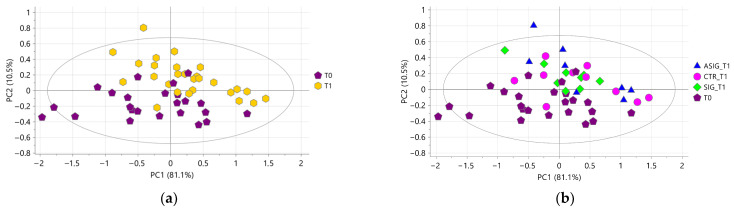
PCA score plot performed on milk aqueous extracts. In (**a**) samples are coloured according to the time of collection (purple pentagons for T0, and yellow hexagons for T1); in (**b**) samples collected at T1 are coloured according to the treatment (blue triangles for ASIG, pink dots for CTR, and green diamonds for SIG) and samples collected at T0 are represented with purple pentagons.

A preliminary PCA performed on all aqueous milk extracts resulted in three components explaining 96.5% of the total variance, with Q^2^ = 65.1%. After excluding three strong outliers, a new PCA was performed characterized by three components explaining 94.7% of the total variance, with Q^2^ = 47.5%. The score plot showed a natural clustering of samples according to the sampling time points (Figure 3a) along with a complete overlap of the samples belonging to the three treatments at **T1** (Figure 3b). These results indicate that the aqueous milk extracts were strongly influenced by the physiological stages of the cow’s life and not influenced by the treatment administered.

**Figure 4:**
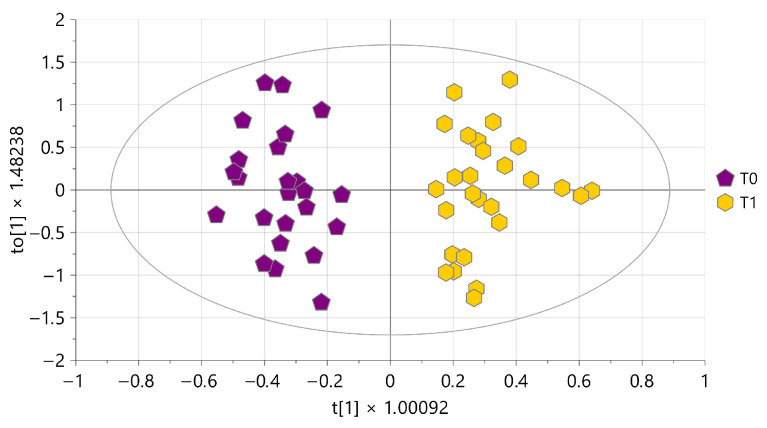
OPLS-DA score plot performed on milk aqueous extracts. Samples are coloured according to the time of collection: purple pentagons for T0, and yellow hexagons for T1. R^2^X = 93.3%, R^2^Y = 88.6%, and Q^2^ = 84.1%. Permutation test resulted in Q^2^ < 0 and R^2^Y = 0.05.

A two-class OPLS-DA model was performed to highlight the variables responsible for the natural clustering of samples according to the collection time point. The model consisted of one predictive and two orthogonal components (Figure 4), showing a clear-cut separation of samples along the first predictive component. The corresponding S-plot (Figure S2) indicated that milk samples at **T0** were characterized by a higher content in choline (buckets 3.21–3.23 ppm and 4.04–4.07 ppm), butyrate (buckets at 0.62–1.04 ppm and 2.11–2.20 ppm), leucine (buckets at 0.62–1.04 ppm and 1.61–1.76 ppm), valine (buckets at 0.62–1.04 ppm and 2.24–2.32 ppm), and N-Acetylglucosamine and 9, N-acetylcarbohydrates (bucket 2.04–2.09 ppm).

**Figure 5:**
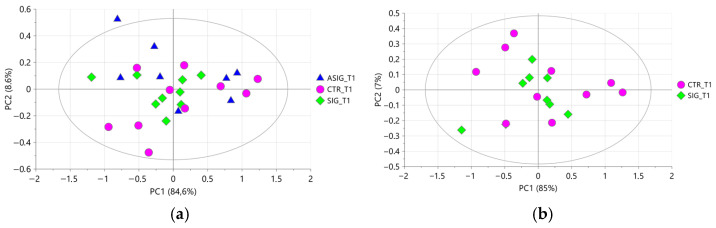

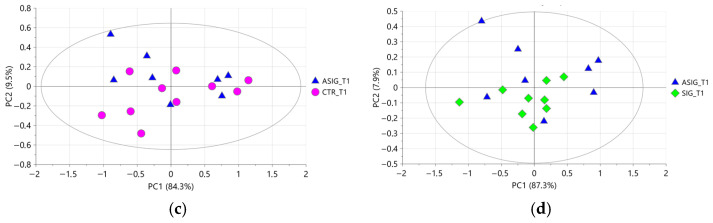
PCA score plots performed on all (**a**), CTR and SIG (**b**), CTR and ASIG (**c**), and SIG and ASIG (**d**) of aqueous milk extracts collected a T1. Samples are colored according to the treatment: pink dots for CTR, blue triangles for ASIG, and green diamonds for SIG. (**a**) 2PCs, R^2^X = 93.2% and Q^2^ = 55%, (**b**) 2PCs, R^2^X = 92% and Q^2^ = 29.1%, (**c**) 2PCs, R^2^X = 93.8% and Q^2^ = 47.3%, and (**d**) 4PCs, R^2^X = 99% and Q^2^ = 74.4%.

Samples at **T1** showed a greater amount of saccharide moieties (bucket at 3.52–3.99 ppm), citrate (buckets 2.49–2.55 ppm and 2.66–2.72 ppm), phosphorylcholine (buckets 3.23–3.24 ppm and 4.16–4.23 ppm), and galactose-1-phosphate (bucket at 5.51–5.56 ppm). Successively, the analysis focused exclusively on the samples collected at **T1** to assess whether the treatment induced sample differentiation at this time point. PCA conducted on all samples, as well as on subsets of milk samples from cows subjected to pairwise treatment comparisons, did not reveal any treatment-related clustering (Figure 5a–d) and the corresponding PLS-DA models did not fit. The findings demonstrate that the use of internal teat sealant alone or in combination with oral supplementation of *Aloe arborescens* did not alter the metabolite composition of the aqueous extracts. The metabolite profiles were comparable between these treatments and, notably, also to those observed in milk from cows subjected to the conventional dry-off procedure involving antibiotic treatment and the use of an internal teat sealant. Therefore, from a metabolite composition perspective, antibiotic treatment could be successfully replaced by using an internal teat sealant alone, without the need for dietary supplementation of *Aloe arborescens*, at least at the time point considered.

#### 3.2.2. Organic Milk Extract

**Figure 6:**
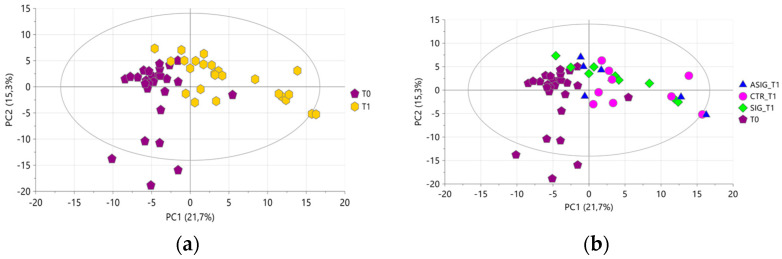
PCA score plot performed on milk organic extracts. In (**a**) samples are coloured according to the time of collection (purple pentagons for T0, and yellow hexagons for T1); in (**b**) samples collected at T1 are coloured according to the treatment (blue triangles for ASIG, pink dots for CTR, and green diamonds for SIG) and samples collected at T0 are represented with purple pentagons.

The PCA performed on all organic milk extracts resulted in eight components explaining 74.5% of the total variance, with Q^2^ = 26.5%. After excluding two strong outliers, a new PCA was performed yielding in eight components explaining 70.1% of the total variance, with Q^2^ = 13.9%. As shown in the score plot reported in Figure 6a, a natural clustering of samples according to the collection time point was evident along with a complete overlap of samples belonging to the three treatments a **T1** (Figure 6b). These data indicate that the metabolite profiles of the organic extracts were also largely influenced by the physiological stages of the cows rather than by the treatment applied to prevent infection diseases, consistent with what was observed in the analysis of the aqueous milk extracts.

**Figure 7:**
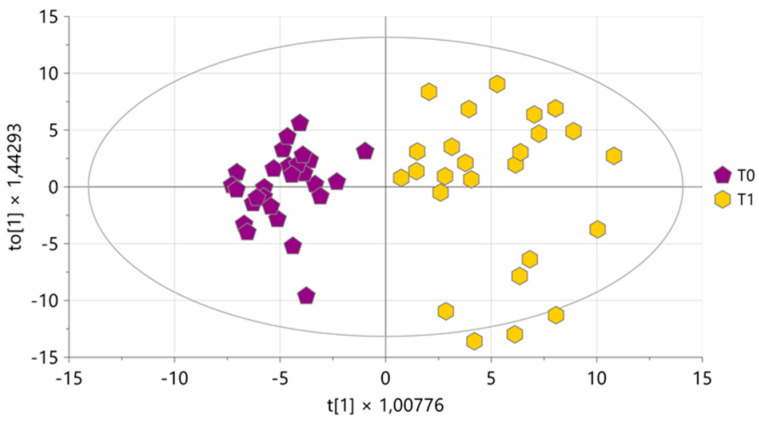
OPLS-DA score plot performed on organic milk extracts. Samples are colored according to the time of collection: purple pentagons for T0, and yellow hexagons for T1. R^2^X = 40.4%, R^2^Y = 84.2%, and Q^2^ = 62.6%. Permutation test resulted in Q^2^ < 0 and R^2^Y = 0.52.

A two-class OPLS-DA model was performed to highlight the organic metabolites responsible for sample differentiation according to the collection time point. The model consisted of one predictive and two orthogonal components (Figure 7). A clear separation of the samples was observed with a wider dispersion for milks collected at **T1**, most likely due to individual metabolic responses of each cow during transition period, which may have affected the metabolite composition.

The corresponding S-plot indicated that milk samples collected at **T0** were characterized by a higher content of CLA 18:2 9-*trans*, 11-*trans* (bucket 5.93–5.99 ppm) and CLA 18:2 9-*cis*, 11-*trans* (bucket 5.86–5.93 ppm and 6.20–6.32 ppm) along with caproleic acid (buckets 5.70–5.83 ppm), linoleic/linolenic, CLA and caproleic acids (buckets from 2.03 to 2.06 ppm) compared with samples collected at **T1**. This finding is consistent with Cremonesi et al., who reported no significant differences in fatty acids content between milk collected before (14 days before dry-off) and after (28 days after calving) the oral administration of *Aloe arborescens*, except for a slightly lower content in CLA 18:2 (9-*cis*, 11-*trans*) and caproleic acid after calving [23].

**Figure 8:**
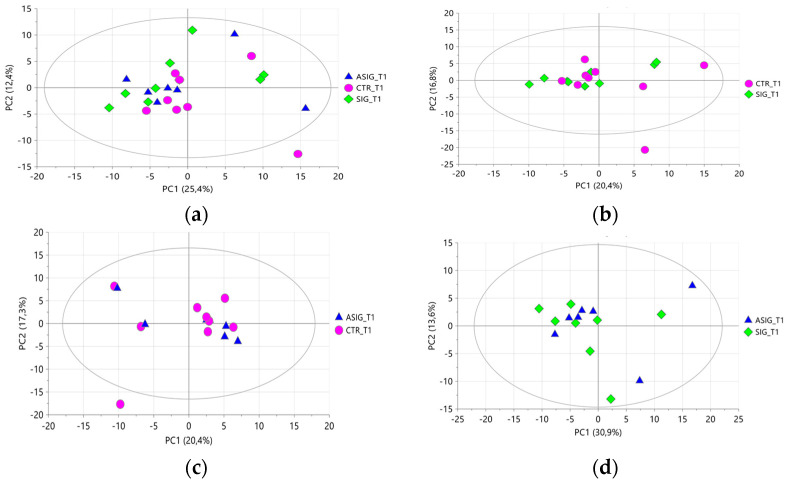
PCA score plots performed on all (**a**), CTR and SIG (**b**), CTR and ASIG (**c**), and SIG and ASIG (**d**) of organic milk extracts collected a T1. Samples are coloured according to the treatment (pink dots for CTR, blue dots for ASIG, and green dots for SIG). (**a**) 4PCs, R^2^X = 53.2% and Q^2^ = 10.5%, (**b**) 4PCs, R^2^X = 58.6% and Q^2^ = negative, (**c**) 3PCs, R^2^X = 50.8% and Q^2^ = negative, and (**d**) 2PCs, R^2^X = 44.5% and Q^2^ = 14.7%.

A deeper investigation into a possible differentiation of samples according to the treatment was performed by considering only the milk samples collected at **T1**, also for the organic extracts. Even in this case, the PCA did not reveal any clustering of samples according to the treatments, either when considering all samples together or when evaluating only the samples obtained from cows subjected to pairwise treatment comparisons (Figure 8a–d). The corresponding PLS-DA models did not fit, indicating that differentiation of samples based on treatment was not achievable.

Interestingly, the findings obtained on the organic extracts confirmed those observed in the aqueous ones: from the perspective of metabolite profiling, antibiotic treatment could be effectively replaced by the use of an internal teat sealant alone, without the need for dietary supplementation with *Aloe arborescens*, at least at the time point considered. Indeed, the organic metabolite composition of milk from cows subjected to the three different treatments, aimed at maintaining physiological well-being, was comparable.

The results are consistent with those of Cattaneo et al., who reported that the *Aloe arborescens* treatment did not lead any difference between groups in the investigated periods in milk composition [19].

## 4. Conclusions

^1^H NMR metabolomics, combined with chemometric analysis, provides a powerful tool to elucidate the metabolite composition of cow’s milk and evaluate the effects of nutritional supplementation and management strategies. The results of the present study indicated that neither intramammary antibiotic administration nor dietary supplementation with *Aloe arborescens* induced significant alterations in metabolite profiles of aqueous and organic milk extracts compared with cows treated with the internal teat sealant alone.

These findings suggest that, from a metabolite perspective, using a physical barrier alone may be the preferable strategy, particularly in antibiotic-restricted production systems, for promoting dairy cow well-being. Alternative interventions are generally associated with higher costs and may be less favorable in terms of animal welfare.

## Data Availability

The data presented in this study are available on request from the corresponding authors.

## Supplementary Materials

The following supporting information can be downloaded at: https://www.mdpi.com/article/10.3390/foods15040770/s1, Figure S1: ^1^H-^1^H TOCSY NMR spectrum (expanded region) of cow milk organic fraction at 600 MHz, in CDCl_3_ at 298 K. The characteristic high field resonances of Cyclopropane fatty acid were shown. Figure S2. S-plot of OPLS-DA score plot performed on milk aqueous extracts. R^2^X = 93.3%, R^2^Y = 88.6%, and Q^2^ = 84.1%. The numbers indicate the initial ppm of each bucket.
