# Supplementary material for: NMR Profiling of Milk from Treated Dried off Cows
Source: Foods. 2026 Feb 20;15(4):770. doi: 10.3390/foods15040770 (PMC12939868; doi:10.3390/foods15040770)
Supplement: Supplementary file 1 [file foods-15-00770-s001.zip › foods-4151249-supplementary.pdf]

Supplementary materials

NMR profiling of milk from treated dried off cows

Antonella Caterina Boccia*, Laura Ruth Cagliani*, Dalila Iannone, Roberto Consonni

National Research Council, Institute of Chemical Sciences and Technologies "G. Natta" (SCITEC), via A. Corti 12, 20133, Milan, Italy

* Correspondence: antonella.boccia@scitec.cnr.it, lauraruth.cagliani@scitec.cnr.it

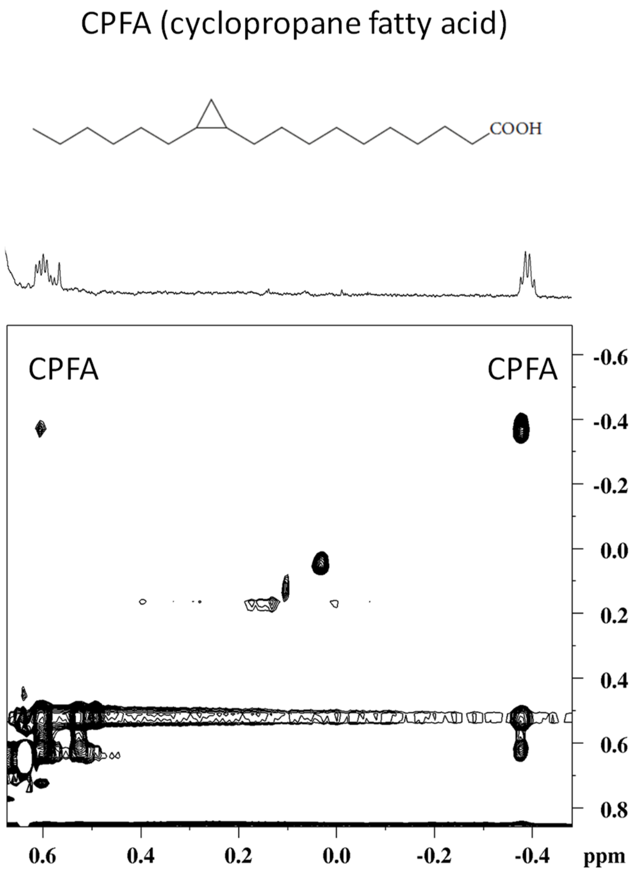

Figure S1: ^1H-^1H TOCSY NMR spectrum (expanded region) of cow milk organic fraction at 600 MHz, in $CDCl_3$ at 298K. The characteristic high field resonances of Cyclopropane fatty acids were showed.

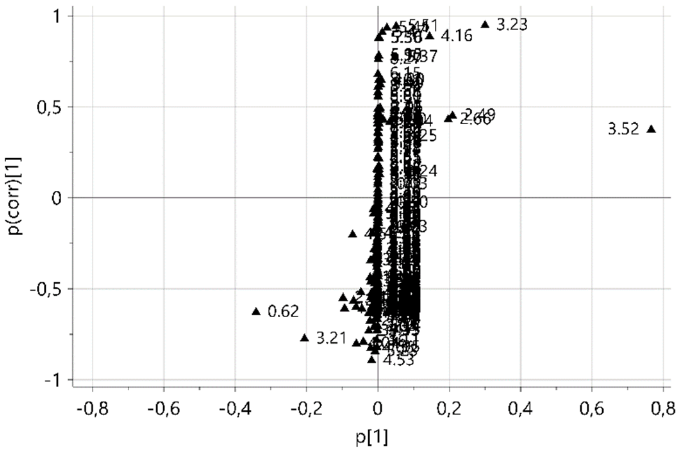

Figure S2. S-plot of OPLS-DA score plot performed on milk aqueous extracts. R^2X=93,3%, R^2Y=88,6%, and Q^2= 84,1%. The numbers indicate the initial ppm of each bucket.
